# Towards Accurate Genotype–Phenotype Correlations in the CYP2D6 Gene

**DOI:** 10.3390/jpm10040158

**Published:** 2020-10-08

**Authors:** Angel L. Pey

**Affiliations:** Departamento de Química Física, Unidad de Excelencia de Química aplicada a Biomedicina y Medioambiente, Facultad de Ciencias, Universidad de Granada, 18071 Granada, Spain; angelpey@ugr.es

**Keywords:** drug metabolism, polymorphism, genotype–phenotype correlations, cytochrome P450

## Abstract

Establishing accurate and large-scale genotype–phenotype correlations and predictions of individual response to pharmacological treatments are two of the *holy grails* of Personalized Medicine. These tasks are challenging and require an integrated knowledge of the complex processes that regulate gene expression and, ultimately, protein functionality in vivo, the effects of mutations/polymorphisms and the different sources of interindividual phenotypic variability. A remarkable example of our advances in these challenging tasks is the highly polymorphic CYP2D6 gene, which encodes a cytochrome P450 enzyme involved in the metabolization of many of the most marketed drugs (including SARS-Cov-2 therapies such as hydroxychloroquine). Since the introduction of simple activity scores (AS) over 10 years ago, its ability to establish genotype–phenotype correlations on the drug metabolizing capacity of this enzyme in human population has provided lessons that will help to improve this type of score for this, and likely many other human genes and proteins. Multidisciplinary research emerges as the best approach to incorporate additional concepts to refine and improve such functional/activity scores for the CYP2D6 gene, as well as for many other human genes associated with simple and complex genetic diseases.

The application of next-generation sequencing technologies is revealing the existence of a vast number of genetic variants in human population, although our ability to reliably identify and quantify the phenotypic consequences and potential pathogenicity of such variants is still quite limited [1,2,3,4,5,6,7]. In some cases, such as non-sense and frame-shift mutations, large deletions and insertions, or duplications, the phenotypic consequences and pathogenicity of these mutations can be inferred more or less straightforwardly. However, and not surprisingly, due to the lower output of functional analyses compared to that of large-scale new-generation sequencing analyses, about half of the currently identified genetic variants are classified as of uncertain significance [2]. This interpretation is further challenged by the large number (about 50%) of these variants identified in only a single individual [8].

A particularly challenging case is the evaluation of the phenotypic consequences of missense mutations. Good correlations between the effects of missense mutations on molecular function and their pathogenic manifestations are sometimes observed, although these correlations are complex in nature (life is intrinsically complex) [1,2,3,9,10]. At the simplest level, a missense mutation can simultaneously affect different functional traits in a given protein, including enzyme kinetics, intracellular stability, subcellular targeting, aggregation propensity and interactions with small molecules and other biomacromolecules [3,9,11,12]. Even at this simple level, the characterization of these molecular effects and their correlation with a given molecular or cellular genotype is challenging, even when protein structural information is available [9]. Remarkably, a good correlation between the effect of missense mutations on protein structural stability, the levels of functional protein in the cell and particular loss-of-function phenotypes has been drawn in some cases [3,7,13,14]. The phenotypic manifestation of mutational effects on molecular functionality also depends on many other factors, such as the genetic and cellular context, environmental factors, stochastic variations, transgenerational transmission of epigenetic information and post-translational modifications (PTMs) [2,3,15,16,17,18]. A paradigmatic example of this mechanistic complexity are those isogenic individuals who manifest widely different disease susceptibilities [19,20].

The *CYP2D6* gene is a very well-documented case in which large genetic variability is known to affect patient’s phenotype in a complex manner [21]. This gene encodes for the cytochrome P450 2D6 monooxygenase, an enzyme involved in the metabolism of a wide variety of biomolecules (such as lipids) as well as several of the most marketed drugs, including SARS-CoV-2 therapies (e.g., chloroquine and hydroxychloroquine) [22,23,24,25]. This gene displays a large genetic diversity among human population, including non-sense, frame-shift and missense mutations and gene duplications [21,26,27]. Despite the inherent complexity in establishing genotype–phenotype correlations in the *CYP2D6* gene, a simple phenotypic score was proposed in 2008 that qualitatively ranked the phenotype (i.e., poor, intermediate, extensive-normal or ultrarapid metabolizers) by assigning a given value for each allele, including activity enhancement due to gene duplications [28]. As these authors recently reviewed, this score has become popular and rather successful, although its simplicity provides an inherent lack of (quantitative) predictive power. This limitation is highlighted, for instance, by the strong interindividual differences in the phenotype within a given genotype or the variable phenotypic manifestation, depending on the drug investigated [21]. This has led to additional factors being proposed that may improve the performance of such a predictive tool for genotype–phenotype correlations in the *CYP2D6* gene such as the presence of alterations (e.g., polymorphisms) in DNA regulatory elements, modulation of *CYP2D6* gene expression by changes in the activity of transcription factors and small, non-coding RNAs, competing effects of substrates and inhibitors of the enzyme and many others [21]. Several additional factors may contribute to improving genotype–phenotype correlations for the *CYP2D6* gene, such as considering the effects of missense mutations on protein structure, energetics and dynamics, or the potential regulatory roles of post-translational modifications (PTMs). First, missense mutations may affect the structure and stability of the protein, leading to changes in the intracellular stability and specific activity. To implement these effects into an activity score for the *CYP2D6* gene, currently available crystal structures for the protein (over 25 are available at the *Protein Data Bank*, including those with bound substrates and inhibitors) [24,29] and suitable structure-based energy force fields could be used for either the prediction of deleterious effects of missense variants detected in human population or for in silico saturation mutagenesis to identify regions that are particularly sensitive to these mutational effects (i.e., hot-spots) [3,7,14,30]. These analyses can be cross-correlated with functional information from expression analysis combined with biochemical and biophysical studies of mutant variants [31]. Second, detailed structural analyses on the CYP2D6 enzyme have supported the existence of a certain level of structural malleability of the protein in adapting its conformation specifically to the presence of substrates and inhibitors [24]. Thus, it is plausible that missense mutations may affect this dynamic behavior of the protein, causing subsequent functional (i.e., enzymatic) alterations. Regarding PTMs, there are currently 10 sites of PTMs identified in the CYP2D6 enzyme by proteomic analyses, although no information is available for the effects of these site-specific PTMs on the function and stability of the protein [32,33]. Six of these correspond to ubiquitinylation events, with potential effects on the stability or regulation of the protein, whereas three of them correspond to phosphorylation sites and one to an acetylation event. Therefore, it is plausible that the complex dynamics underlying these PTMs could lead to different phenotypic manifestations of a given phenotype in different individuals.

Clearly, an increased knowledge of the mechanisms underlying genotype–phenotype correlations associated with the genetic variability of the CYP2D6 will improve our predictive power on the metabolizing function of CYP2D6. The lessons to be learned from this gene could be more generally useful for the accurate prediction of these genotype–phenotype correlations (particularly for missense mutations) on a genome-wide scale, and the implications for individual response to pharmacological treatments, one of the holy grails of Personalized Medicine.
